# Middle Colic Artery Originating from the Gastroduodenal Artery Discovered during a Whipple

**DOI:** 10.1155/2019/1986084

**Published:** 2019-02-11

**Authors:** Mei Li M. Kwong, Jeffrey Pelton

**Affiliations:** ^1^MedStar Washington Hospital Center, Department of General Surgery, Washington, D.C., USA; ^2^Kaiser Permanente, Surgical Oncology, Rockville, Maryland, USA

## Abstract

Hepatic artery variations occur in about one-fourth of the population, are frequent questions on standardized surgery exams, and are often discussed on teaching rounds with an attending surgeon. Intraoperatively, it is important to be aware of possible vascular anomalies in order to prevent ligation or injury of an essential vessel. This case report describes an extremely rare vascular anomaly encountered during a pancreaticoduodenectomy (Whipple operation). Our patient was a middle-aged woman who was incidentally diagnosed with a cystic pancreatic lesion. During the operation, an aberrant middle colic artery was found to be originating from the gastroduodenal artery instead of its usual origin at the superior mesenteric artery. This anomalous middle colic artery has not been previously reported in a live patient. It underscores the importance of being aware of possible vascular variations that may be encountered intraoperatively in order to prevent morbidity and mortality.

## 1. Introduction

Anomalous vascular anatomy during hepaticopancreaticobiliary operations is common. Considering that one-fourth of the patients could have an anomalous hepatic artery and that laparoscopic cholecystectomy is one of the most frequent general surgery operations in the United States, failure to recognize the presence of an anomalous artery could have devastating consequences. Although extremely rare, other vascular anomalies in this region do occur. We report a case of a patient found to have an anomalous middle colic artery arising from the gastroduodenal artery during a pancreaticoduodenectomy. The intraoperative discovery of the middle colic artery originating from the gastroduodenal artery is a unique finding. To our knowledge, this is the first occurrence in a live patient. It underscores the importance of being aware that vascular anomalies in this area occur. A competent surgeon must be diligent and knowledgeable in order to avoid morbidity and mortality by accidently damaging vital structures.

## 2. Case Presentation

We report a case of a middle colic artery originating from the gastroduodenal artery found during a pancreaticoduodenectomy for a pancreatic cystic mucinous neoplasm. The middle-aged patient had asthma and well-controlled hypertension but was otherwise healthy. The cystic lesion at the head of the pancreas was incidentally found two years prior when she had gone to the emergency room with complaints of nausea and abdominal pain. Computer tomography at that time demonstrated a 1.9 × 1.3 cm area of cystic change within the pancreatic head without evidence of pancreatitis. A follow-up MRI of the abdomen characterized the lesion as an intraductal pancreatic mucinous neoplasm. She was initially observed since the abdominal pain had resolved and there were no worrisome features of the pancreatic cystic lesion.

On repeat imaging a year later, the mass was visualized to be in communication with the nondilated pancreatic duct and had grown to 3 cm in size. The lesion was further characterized by endoscopic ultrasound. It demonstrated a 3.1 × 2.6 cm multicystic and septated anechoic structure in the pancreatic head and uncinate process. Mucoid material was aspirated, and pathology demonstrated minimal cellularity without malignant cells. The amylase and carcinoembryonic antigen levels of the aspirate were elevated at 6769 units/L and 10,563 units/L, respectively. It was decided that she should undergo surgical resection. The location of the tumor and involvement with the main pancreatic duct required a pancreaticoduodenectomy. The review with radiology of preoperative imaging did not reveal any vascular anomalies.

**Figure 1:**
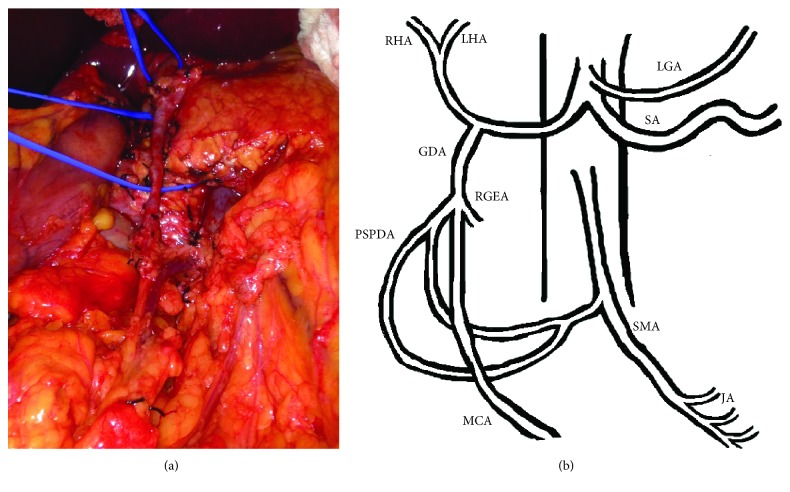
Middle colic artery arising from the gastroduodenal artery. (a) Intraoperative photograph. One vessel loop is around the neck of the pancreas, and the second vessel loop is encircling the origin of the gastroduodenal artery. The anterior and posterior superior pancreaticoduodenal arteries and smaller unnamed branches off the gastroduodenal artery have been ligated. The common bile duct has already been transected. (b) Illustration of the middle colic artery arising from the gastroduodenal artery as seen during the pancreaticoduodenectomy. RHA: right hepatic artery; LHA: left hepatic artery; LGA: left gastric artery; SMA: superior mesenteric artery; SA: splenic artery; RGEA: right gastroepiploic artery; PSPDA: posterior superior pancreaticoduodenal artery; MCA: middle colic artery; JA: jejunal arteries.

**Figure 2:**
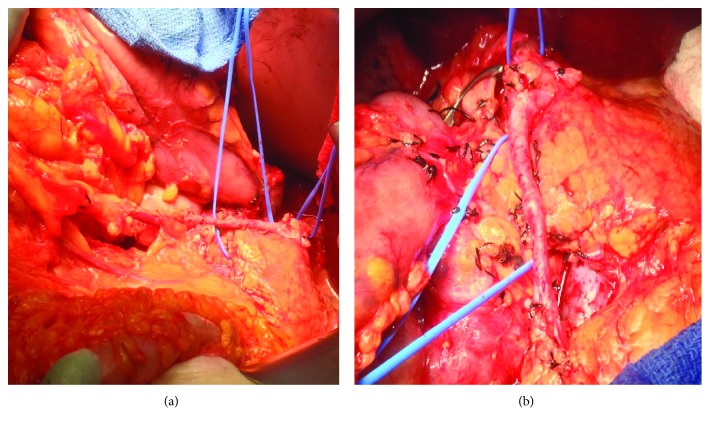
Middle colic artery arising from the gastroduodenal artery. Intraoperative photographs. One vessel loop is around the neck of the pancreas, and the second vessel loop is encircling the origin of the gastroduodenal artery. The anterior and posterior superior pancreaticoduodenal arteries and smaller unnamed branches off the gastroduodenal artery have been ligated. The common bile duct has already been transected. (a) The aberrant middle colic artery can be seen running into the transverse colon mesentery (transverse colon seen in the bottom left corner). (b) The aberrant middle colic is seen crossing on top of the pancreatic neck. The common bile duct is transected, and the bull dog clamp is on the proximal common bile duct.

At exploratory laparotomy, the gastroduodenal artery was identified and dissected out. However, the artery was noted to be very large and continued to traverse across the neck of the pancreas anteriorly down to the root of the mesentery. The middle colic vein was traced to the superior mesenteric vein. The inferior border of the pancreas was mobilized using the harmonic scalpel. Further meticulous dissection of the aforementioned vessel was now performed, and it was traced into the mesentery of the transverse colon, taking the usual course of the middle colic artery. Considerable time was taken carefully ligating small branches of this vessel into the pancreas using a 3-0 silk suture to completely free the vessel off of the neck of the pancreas and preserve it. The pulse of the middle colic artery in the mesentery was then carefully palpated. The anomalous vessel was then occluded, and the pulse within the transverse mesocolon disappeared. The pulse returned to the transverse mesocolon when pressure on the anomalous vessel was released. This indicated that the vessel was an anomalous middle colic artery that originated from the gastroduodenal artery (Figures 1 and 2). The pancreaticoduodenectomy was performed safely, and this vessel was preserved. The patient recovered uneventfully and was discharged home on postoperative day ten.

## 3. Discussion

The intraoperative discovery of the middle colic artery originating from the gastroduodenal artery is a unique finding. Since routine ligation of the gastroduodenal artery is an essential step during pancreaticoduodenectomy, the identification and preservation of this vascular anomaly prevented ischemic and possibly catastrophic injury to the transverse colon. Review of the surgical literature reveals a single case report of this vascular variation found in two cadavers [1]. Other origins of the middle colic artery reported include the splenic artery, common hepatic artery, inferior mesenteric artery, inferior pancreaticoduodenal artery, and aorta or an origin in combination with right colic and/or ileocolic arteries [1–4]. To our knowledge, this is the first and only case of this vascular anomaly discovered during surgery. It is well known that this area of vascular anatomy is the most anomalous vasculature in the human body; therefore, it is not entirely surprising that anomalies such as that reported here may occur.

**Figure 3:**
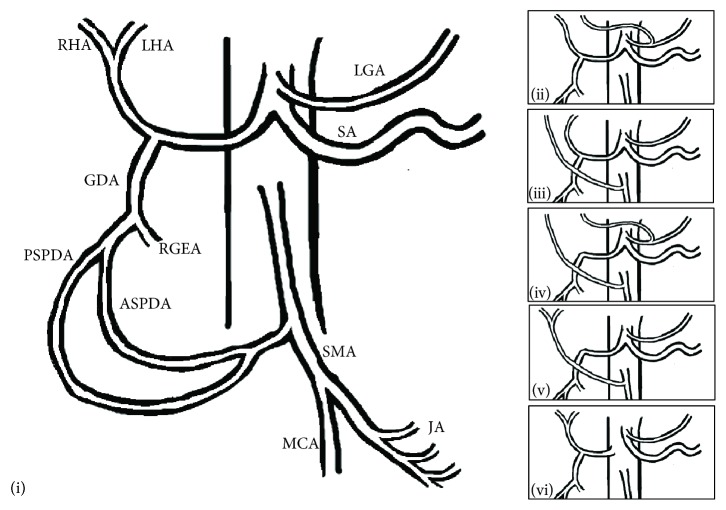
Hiatt's classification of hepatic arteries. Illustrations of Hiatt's types I-VI. RHA: right hepatic artery; LHA: left hepatic artery; LGA: left gastric artery; SMA: superior mesenteric artery; SA: splenic artery; RGEA: right gastroepiploic artery; PSPDA: posterior superior pancreaticoduodenal artery; ASPDA: anterior superior pancreaticoduodenal artery; MCA: middle colic artery; JA: jejunal arteries.

Since Michels' 1966 publication of an internationally recognized classification system of 10 hepatic artery variations based on 200 cadavers, there have been other reports of rare celiac artery variations [5–7]. Hiatt et al. simplified the classification of hepatic artery variations to six main types (Figure 3) [6]. Type I is the most common with right and left hepatic arteries arising from the common hepatic artery that comes off the celiac trunk. Type II has a replaced or accessory left hepatic artery from the left gastric artery. Type III has a replaced or accessory right hepatic artery off the superior mesenteric artery. Type IV has both right and left replaced and/or accessory hepatic arteries. Types V and VI have the common hepatic artery arising from the superior mesenteric artery and aorta, respectively.  Table 1 describes Hiatt's system and the incidence found during angiography based on his and Koops' retrospective data of 1000 and 604 patients, respectively [6, 7].

This case report describes only one patient with this vascular anomaly, and it is unlikely that this vascular anomaly will be encountered during the average hepatobiliary surgeon's career. However, this finding highlights the importance of careful dissection and cognizance of the frequency of anomalous vasculature in this area so as to avoid iatrogenic injury during surgery. It is possible that other vascular anomalies in this anatomic area exist, as yet undescribed.

## Figures and Tables

**Table 1:** Hiatt's classification of hepatic arteries. The table lists Hiatt's classification of hepatic arteries including the description and percentages as reported by Hiatt and Koops.

Type	Description	Percentage (%)
I	Normal: RHA and LHA arising from CHA off the celiac trunk	75.7–79.1
II	Replaced/accessory LHA off LGA	3.0–9.7
III	Replaced/accessory RHA off SMA	10.6–11.9
IV	Both replaced/accessory RHA and LHA	1.3–2.3
V	CHA off SMA	1.5–2.9
VI	CHA off the aorta	0.2

RHA: right hepatic artery; LHA: left hepatic artery; LGA: left gastric artery; SMA: superior mesenteric artery; SA: splenic artery; RGEA: right gastroepiploic artery; PSPDA: posterior superior pancreaticoduodenal artery; ASPDA: anterior superior pancreaticoduodenal artery; MCA: middle colic artery; JA: jejunal arteries.
